# Cognitive screening tools for primary care settings: examining the ‘Test Your Memory’ and ‘General Practitioner assessment of Cognition’ tools in a rural aging population in Greece

**DOI:** 10.1080/13814788.2017.1324845

**Published:** 2017-06-12

**Authors:** Eliza Iatraki, Panagiotis G. Simos, Antonios Bertsias, George Duijker, Ioannis Zaganas, Chariklia Tziraki, Alexandros N. Vgontzas, Christos Lionis, on behalf of the THALIS Primary Health Care Research Team/Network

**Affiliations:** ^a^ Clinic of Social and Family Medicine, Faculty of Medicine, University of CreteHeraklionGreece; ^b^ Department of Psychiatry, Faculty of Medicine, University of CreteHeraklionGreece; ^c^ Department of Neurology, Faculty of Medicine, University of CreteHeraklionGreece; ^d^ MELEBEV Community Elders Clubs, Research DepartmentJerusalemIsrael

**Keywords:** Cognitive screening, primary care setting, rural population

## Abstract

**Background:** Under conditions of high demand for primary care services in a setting of low financial resources, there is need for brief, easily administered cognitive screening tools for use in the primary care setting, especially in rural areas. However, interpretation of these cognitive tests’ results requires knowledge on their susceptibility to cultural, educational and demographic patient characteristics.

**Objectives:** To assess the clinical validity of the ‘Test Your Memory’ (TYM) and ‘General Practitioner assessment of Cognition’ (GPCog) which was specifically designed for primary care practice, in a rural primary care setting in Greece, utilizing the ‘Mini Mental State Examination’ (MMSE) as a reference standard.

**Methods:** The MMSE, TYM, and GPCog were administered to a random sample of 319 community dwelling Greek adults aged 60 to 89 years in 11 rural Primary Healthcare Centres of the Prefecture of Heraklion on the island of Crete, Greece. Analyses examined (a) The association of each instrument with demographic factors and MMSE and (b) optimal cut-off scores, sensitivity and specificity against MMSE-based cognitive impairment risk using ROC analyses with the MMSE 23/24 point cut-off as a reference standard.

**Results:** We found a sensitivity of 80% and a specificity of 77% for TYM (35/36 or 38/39 cut-off, depending on education). Corresponding values were 89% and 61% for GPCog (7/8 cut-off), respectively.

**Conclusion:** The TYM and GPCog instruments appear to be suitable for routine use in the primary care setting as tools for cognitive impairment risk detection in elderly rural populations.

KEY MESSAGES

- Tools for cognitive screening in rural primary care must be brief, easily scored and relatively unaffected by sociodemographic factors.
- TYM and GPCog scales display fair concordance with MMSE-based determination of cognitive decline risk and may be used with ease in clinical practice for cognitive screening of older adults in Greek rural settings.

## Introduction

Detection of early signs of dementia is crucial and may be missed in a busy primary care practice until functional impairment becomes apparent [1]. The Mini-Mental State Examination (MMSE) remains the most widely used screening tool for cognitive impairment [2,3]. Perhaps the most serious obstacle for incorporating the MMSE into primary care practice concerns the time and special training it requires for administration and scoring [3] However, in primary care, MMSE may not be the best option since an ideal cognitive screening test in this setting should be brief, easily administered and scored, while maintaining adequate sensitivity and specificity [3]. Such tools, which were specifically designed for general medical and primary care settings, are the Test Your Memory Test (TYM) and General Practitioner assessment of Cognition (GPCog) [4,5].

The TYM consists of eleven tasks or scales, assessing specific domains of memory related cognitive function, takes 5–10 min to complete with minimal supervision, and can be easily scored by non-medical personnel [4]. The GPCog-Patient scale is a much shorter instrument that can be completed in less than 4 min by a health professional [5]. Both instruments have been translated into several languages and their clinical validity has been assessed for detecting possible cognitive impairment against other screening instruments (such as the MMSE) and/or clinical diagnosis of dementia [1,4,6]. Since the vast majority of validation studies have been conducted with clinical samples recruited primarily in urban settings, it is crucial to assess the validity of these new instruments for detecting risk for cognitive impairment in rural elderly populations, which are also characterized by limited formal education [7]. This need is supported by considerable suggested adjustments to MMSE cut-offs [7–10], potentially increasing false-negative rates in detecting dementia.

Whereas information on the validity of the Greek version of TYM has been reported previously (in a neurology clinic sample) [11], such data is lacking for GPCog. Populations, such as those living in rural regions in Greece, include a very high proportion of persons who have completed fewer than six years of formal education and their capacity to cope with complex instructions and unfamiliar tasks may lead to an overestimation of cognitive impairment risk [12]. The present study extends previous reports from populations with similar characteristics in other European countries regarding the development of primary care and struggle from the economic crisis (e.g. Portugal and Italy) [7,10], which were based primarily on the MMSE, and was designed to assess the clinical validity of two relatively novel cognitive screening tools (TYM and GPCog) in a largely rural, community dwelling sample of Greek elders.

## Methods

### General design, participants and data collection

The sample included 319 community dwelling elders aged 60 to 89 years who were randomly selected from a larger epidemiological cohort (*n* = 3140) of visitors in 14 PHC units (11 located in rural and semi-urban areas) in the Prefecture of Heraklion on the island of Crete, Greece (Cretan Aging Cohort). The overreaching project objective was to assess the burden of cognitive impairment among Cretan community dwelling elders and establish demographic, lifestyle, genetic, psycho-emotional, sleep, and inflammatory correlates of age-related cognitive decline and took place between March 2013 and May 2014. All PHC visitors aged ≥60 years were invited to participate by their GP. The reason for visit was mainly (90%) prescription renewal and response rate was 92.2%. Other than their willingness to participate in the study, no other exclusionary criteria were implemented.

The present sample included 64.6% women, retirees from manual labour professions (73%), and persons who had not attended high school (88.8%; Tables 1 and 2) and was comparable to the overall cohort on the percentage of persons performing manual labour professions (74.8%, *P* = 0.2) and persons who did not attend secondary education (83.1%, *P* = 0.09), but included a higher percentage of women (56.2%; *P* = 0.007). A specially trained study nurse assessed sociodemographic factors and medical history in the participants’ native language (Greek) during a face-to-face interview, who also administered the MMSE, TYM, and GPCog instruments in random order across participants.

**Table 1.** Community sample demographics by cognitive impairment risk group.

	Entire sample (*n* = 319)	MMSE ≤23 (*n* = 81)	MMSE ≥24 (*n* = 238)
Gender, *n* (%)^b^			
Women	206 (64.6)	61 (75.6)	145 (61.0)
Men	113 (35.4)	20 (24.4)	93 (39.0)
Age (mean ± SD in years)^a^	71.0 ± 6.9	74.2 ± 7.0	70.1 ± 6.5
Age groups, *n* (%)^a^			
60–69	153 (48.3)	24 (29.5)	129 (54.4)
70–79	123 (38.6)	36 (44.9)	87 (36.5)
80–89	43 (13.2)	21 (25.6)	22 (9.1)
Education (mean ± SD in years)^a^	6.4 ± 3.1	4.8 ± 2.5	6.8 ± 3.1
Education groups, *n* (%)^c^			
0–6	261 (81.8)	75 (92.2)	186 (78.3)
7–12	48 (15.1)	6 (7.7)	42 (17.5)
≥13	10 (3.1)	–	10 (4.2)
Past occupation, *n* (%)^d^			
Farmer	122 (38.1)	24 (29.8)	98 (40.6)
Labourer	27 (8.6)	3 (4.3)	24 (9.4)
Housekeeper	84 (26.3)	33 (40.4)	51 (23.1)
Clerical	44 (13.7)	5 (6.4)	39 (15.0)
Technician	15(4.7)	4 (4.3)	11 (4.7)
Educator	2 (0.7)	–	2 (0.9)
Business owner	25 (7.9)	12 (14.9)	13 (6.4)
Family status, *n* (%)			
Single	6 (1.8)	3 (3.4)	3 (1.3)
Married	231 (72.3)	50 (61.8)	181 (75.8)
Widowed/divorced	82 (25.9)	28 (34.8)	54 (22.9)

^a^ *P* <0.0001,

^b^ *P* = 0.01,

^c^ *P* = 0.015,

^d^ *P* = 0.02.

**Table 2.** Community sample clinical characteristics and performance on MMSE, TYM, and GPCog by cognitive impairment risk group.

	Entire sample (*n* = 319)	MMSE ≤23 (*n* = 81)	MMSE ≥24 (*n* = 238)
Depression (%)	46 (14.5)	17 (21.0)	29 (12.8)
Dementia (%)^a^	12 (3.9)	11 (13.4)	2 (1.3)
PD (%)	3 (1.0)	2 (2.4)	1 (0.7)
CVA (%)	6 (1.8)	3 (3.7)	3 (1.3)
Functionality^a^			
Fully independent (%)	167 (52.5)	51 (62.2)	196 (82.2)
Partly dependent (%)	152 (47.5)	30 (37.8)	42 (17.8)
MMSE			
Mean ± SD	26.0 ± 3.2	21.3 ± 2.0	27.5 ± 1.8
Range	14–30	14–23	24–30
TYM			
Mean ± SD	38.5 ± 7.9	30.5 ± 8.3	41.1 ± 5.7
Range	2–50	2–45	19–50
GPCog			
Mean ± SD	6.8 ± 2.2	4.8 ± 2.4	7.4 ± 1.7
Range	1–9	1–9	2–9

^a^ *P* < 0.0001.

PD: Parkinson’s disease; CVA: cerebrovascular accident.

The study was approved by the Ethics Committee of the University Hospital of Heraklion, Crete and all participants provided written consent following detailed briefing on study purpose and procedures.

### Instruments

#### TYM Test

The *TYM Test* consists of eleven tasks or scales evaluating specific memory functions. The TYM takes 5–10 min to complete with minimal supervision [4]. The maximum score is 50 points (indicating perfect performance). Inter-rater agreement for scoring in the original English version was excellent (99%). The original validation study reported excellent sensitivity (93%) and specificity (86%) using a score of 42 points to differentiate between patients diagnosed independently with mild probable AD and controls [4]. The procedure adopted for the translation and cultural adaptation of TYM in Greek is described in Iatraki et al. [11].

#### GPCog scale

The GPCog-Patient section, which has a maximum score of nine points (indicating perfect performance), was translated and culturally adapted into Greek according to the Minimal Translation Criteria developed by the Scientific Advisory Committee of the Medical Outcomes Trust with written permission from the original developers of the instrument. In the original validation study, sensitivity and specificity against clinical diagnosis of dementia was 85% and 86%, respectively [5].

#### Mini-mental state examination

The MMSE assesses five cognitive domains: orientation, registration, attention and calculation, recall, and language, yielding a total score of 30. In elderly patients’ samples from several Western countries including Greece displaying relatively high levels of literacy, sensitivity and specificity values ranged between 0.70 and 0.90 using the standard cut-off of 23/24 points [2–4].

### Data analysis

The contribution of age, education and gender on TYM, GPCog and for comparison, MMSE test scores was examined through multiple linear regression analyses (all variables were force-entered in the model). Given the low average education level of the sample and the distribution of MMSE scores, the originally suggested cut-off of 23/24 points was employed, resulting in 81 persons (25%) identified as at risk for cognitive impairment [2,13]. This cut-off was used to create the grouping variable against which sensitivity, specificity, Positive Predictive Value (PPV), and Negative Predictive Value (NPV) of each test were estimated through ROC analyses. Optimal cut-off points for TYM and GPCog were determined using the Youden Index defined as the TYM or GPCog score associated with J = max {Sensitivity + Specificity -1} [14].

In addition, the Bland–Altman method was used to assess the compatibility of TYM or GPCog with MMSE [15]. Given that the three tools possess different measurement scales, raw scores were first z-transformed in the entire sample.

## Results

Internal consistency estimates (Cronbach’s alpha) for MMSE, TYM, and GPCog were 0.80, 0.77, and 0.79, respectively. Preliminary analyses confirmed the significant effects of age (negative) and education level (positive) with TYM, GPCog, and MMSE scores. Interestingly, the effect of gender (higher age and education-adjusted scores for men) reached significance only for MMSE.

### Associations between TYM and GPCog with reference standard MMSE scores

The associations between TYM and MMSE (R^2^ = 0.52, β_linear_ = 0.785, *t* = 3.48, *P* = 0.001, β_quadratic_ = –0.066, *t* = –0.29, *P* = 0.77) and between GPCog and MMSE approached linearity (R^2^ = 0.43, β_linear_ = 0.761, *t* = 3.51, *P* = 0.001, β_quadratic_ = –0.108, *t* = –0.50, *P* = 0.61; Table 3). Moderated regression analyses further established that the magnitude of associations between TYM-MMSE and GPCog-MMSE did not vary with education level.

**Table 3.** Associations between demographic characteristics, TYM, MMSE and GPCog total scores. Zero-order Pearson correlations are shown above the diagonal and partial correlations controlling for age and education below the diagonal.

	MMSE	TYM	GPCog
Age	–0.41^a^	–0.45^a^	–0.45^a^
Education	0.39^a^	0.49^a^	0.38^a^
MMSE	1	0.75^a^	0.69^a^
TYM	0.66^a^	1	0.70^a^
GPCog	0.59^a^	0.58^a^	1

^a^ *P* = 0.0001.

### Clinical Validity of TYM and GPCog

The at-risk group (based on MMSE) included more women and older persons who had attained fewer years of formal education (Table 1) and had lower average scores on TYM and GPCog (Table 2).

**Figure 1.**
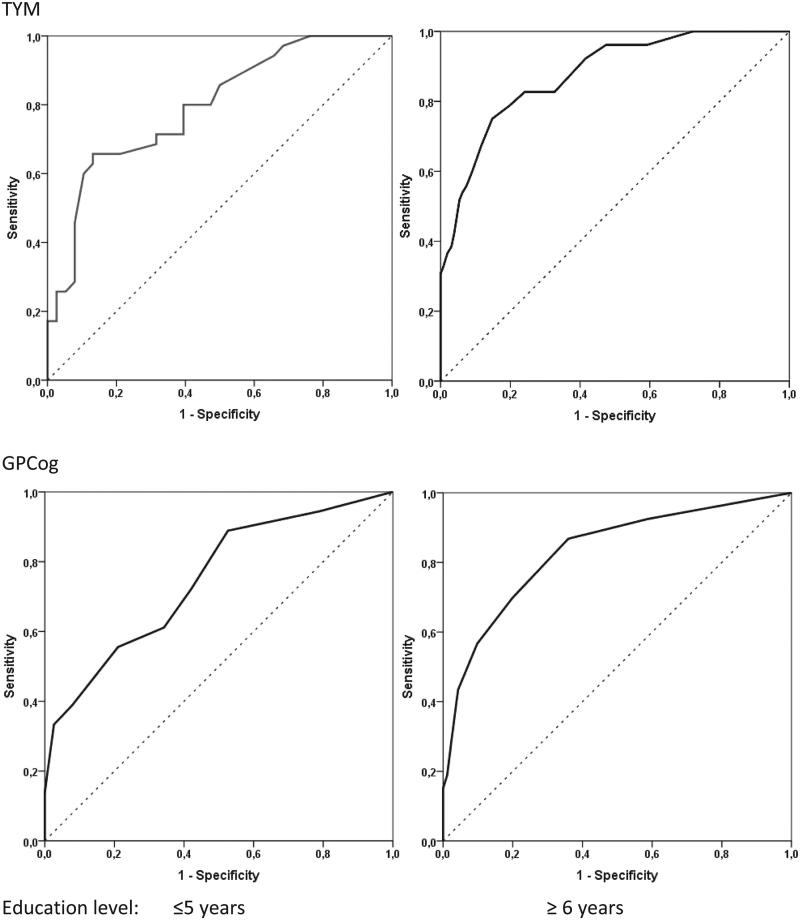
Receiver Operating Characteristic Curves of TYM (upper panel) and GPCog scores (lower panel) for detecting risk of cognitive impairment based on MMSE performance.

The results of the ROC analyses, conducted separately for persons with low (i.e. ≤5 years) and persons with greater education level (≥ 6 years), are summarized in Figure 1. According to the J index, the optimal cut-off value on TYM is 35/36 points for low-educated persons, ensuring a minimum acceptable sensitivity of 80%. The corresponding cut-off for persons with higher education is 37/38 points (associated with 75% sensitivity). To ensure a minimum sensitivity of 80%, we suggest that a slightly higher cut-off value of 38/39 should be adopted.

For GPCog a universal cut-off of 7/8 points was associated with the maximum J index and 80% sensitivity. Overall sensitivity, specificity, PPV, and NPV values in the entire sample using these cut-off scores for each test are displayed in Table 4. The impact of education level on the clinical validity of both tests is further indicated by area under the curve values approaching 0.90 for persons with six or more years of education (indicating high discrimination accuracy) and 0.75–0.79 for persons with lower education (indicating moderate accuracy; Figure 1) [16].

**Table 4.** Sensitivity and specificity for identifying at risk individuals using TYM and GPCog scores against MMSE-defined cognitive impairment risk. Optimal cut-off values associated with maximum J index (Sensitivity + Specificity –1).

	TYM	GPCog
Cut-off	35/36 or 38/39^a^	7/8
Sensitivity	0.80	0.89
Specificity	0.77	0.61
Positive predictive value	0.47	0.38
Negative predictive value	0.93	0.95

^a^ 35/36 for persons with ≤5 years of education and 38/39 for persons with ≥6 years of education.

**Figure 2.**
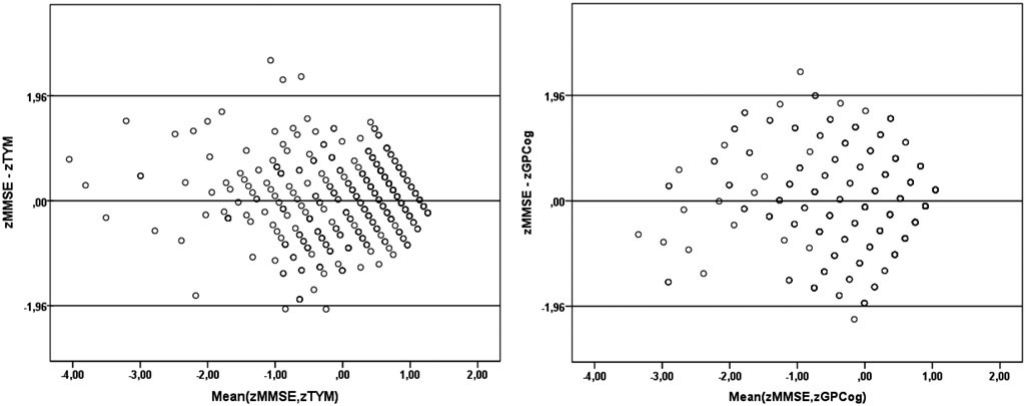
Bland–Altman plots illustrating the distribution of standardized score differences for each pair of tests (MMSE minus TYM in the left-hand panel and MMSE minus GPCog in the right-hand panel) as a function of corresponding average scores.

The average difference of normalized scores between TYM and MMSE (0.0099) and between GPCog and MMSE (–0.0028) was not significantly different from 0 (*P* > 0.7 and *P* > 0.9, respectively), as per Bland–Altman technique. Inspection of the Bland–Altman plots in Figure 2, reveals that the standardized difference for each pair of tests rarely exceeded one standard deviation from the mean (5/314 cases on TYM and 2/314 cases on GPCog). It was further noted that the greatest dispersion of difference scores was found among persons scoring in the low average range on both tests (i.e. within approximately 1 SD below the mean).

## Discussion

### Main findings

Data from this random, rural sample of community dwelling elders indicate that both TYM and GPCog display comparable internal consistency properties to the MMSE, and had fair concordance with the MMSE-based determination of risk for cognitive decline, as indicated by sensitivity of 80% and specificity of 77% for TYM, and corresponding values of 89% and 61% for GPCog.

### Discussion in light of the literature

#### Comparison of TYM and GPCog with MMSE

Sensitivity and specificity estimates were virtually identical to the original TYM standardization study and to those obtained in a clinical sample of Greek elders [11]. Internal consistency estimates for GPCog were also very like the original standardization study and somewhat higher than in other cultures (e.g. French and Chinese) [5,17,18].

Sensitivity and specificity values for each instrument varied noticeably with educational level, highlighting the contribution of additional factors to TYM and GPCog scores, especially for persons with minimal formal education (e.g. literacy level associated with personal interests, occupational experiences and engagement in reading and other cultural activities).

#### Demographic effects

Demographic effects on TYM, GPCog and MMSE were similar with the exception of gender: although men and women displayed comparable scores on TYM and GPCog, age and education-adjusted MMSE scores were higher among men. In our sample *age effects* were modest (*r* = –0.41 to –0.45). The magnitude of the effect of *educational level* on the two instruments was comparable to MMSE (*r* = 0.38 to 0.49) and tended to be stronger for persons with fewer years of schooling. The effect of education level on TYM appeared to be more pronounced (further stressed by the need to implement a substantial correction of cut-off scores for persons with ≤5 years of formal education in order to maintain acceptable clinical validity). This finding may have been largely neglected in previous validation studies.

Educational level and literacy are known determinants of performance on screening instruments and a well-documented risk factor for dementia [19,20], although causality has not been firmly established [21]. Previous reports have been inconclusive regarding the dependence of TYM and GPCog upon demographic variables [22–24]. However, both the range of demographic variables sampled by each study cohort and the scarcity of persons with very few years of formal education may have biased earlier reports.

### Strengths and limitations

The key limitation of the present study is that the clinical validity of TYM/GPCog was assessed through comparison with a screening test that was used to establish risk for cognitive impairment instead of clinical diagnosis of dementia. Although, MMSE is widely used and considered by many as the gold-standard instrument for dementia screening, it has far from perfect sensitivity or specificity [3] In mixed urban/rural samples the Greek version of MMSE (using a 23/24 point cut-off) was associated with 0.70–0.80 sensitivity and 0.62–0.90 specificity [11,13]. Specificity for detecting clinically diagnosed dementia may be considerably lower, however, (40%) in rural elderly populations as suggested by unpublished data (*n* = 505) from the Cretan Aging Cohort (Basta et al., personal communication), in agreement with data from low literacy and/or rural elderly samples in a variety of cultures [7–10]. Future studies should directly compare the three instruments against clinical diagnosis of dementia in the same rural elderly population.

### Implications for clinical practice

TYM and GPCog can be administered by GPs or other health personnel with minimal training and appear to be suitable for primary care settings serving elders with no or minimal formal education [3,25]. They may prove particularly useful in rural areas where there are limited resources, expertise in state-supported primary care systems and accessibility to specialists, such as the case in Greece where, in addition, formal recommendations for screening tool use by primary care physicians are lacking. Only recently general guidelines have been published in Greece in the context of the National Action Plan for Dementia and Alzheimer’s Disease 2015–2020, stressing the importance of developing and using such tools and associated screening procedures in primary care [26].

The limited gender and education-level susceptibility of GPCog render it preferable over TYM for routine use in primary care settings in accordance with a growing number of studies in other cultures [17,18,23]. Additional desirable attributes of GPCog include the following [1]: it requires 5 min or less to administer; it is validated in a primary care or community setting; it is easily administered by medical staff members who are not physicians; it has good to excellent psychometric properties; it is relatively free from educational, language, and/or culture bias; it can be used in a clinical setting without copyright cost [27]. Its capacity to detect cognitive impairment may be further improved if supplemented by the GPCog Informant Scale [28]. However, the final choice of instrument is left to the clinical judgment of the GP.

The generalizability of the current results to other, especially, southern European populations is supported by important historic and sociocultural similarities. Comparable characteristics include similar social structure featuring a prominent role of extended family and severely limited access to adequate quality, systematic basic education during WWII and the following years (due to severe poverty, internal conflicts and devastated state infrastructure and services). Additional similarities in current conditions of rural areas in, especially Mediterranean, European countries include higher percentage of older residents; lower per capita income and higher poverty rate, who face greater obstacles in accessing health services and high quality, specialized care [29,30].

## Conclusion

The TYM and GPCog instruments demonstrate desirable properties for cognitive impairment risk detection in primary care settings, although the former test requires substantial adjustment of cut-off scores according to education level.
